# 

**Published:** 1892-08

**Authors:** 


### CONTENTS.

ORIGINAL ARTICLES—The Radical Cure of Hernia, Particularly with Children—By Thos. H. Manley, A. M., M. D., New York ........... 423
Diphtheria—By A. Bowen, M D., Nebraska City, Neb ............... 433
Turpentine in Typhoid—By J. J Waller, M. D., Oliver Springs, Tenn. 456
Report of a Case—By R. R. Kime, M. D., Atlanta Ga .................. 438
CORRESPONDENCE—Our New York Letter .................................. 442
SOCIETY NOTES—Clinical Society of Maryland ........................... 445
The American Health Resort Association .................................. 452
BOOK REVIEWS ........................................................... 455–457
International Clinic—Transaction of the Southern Surgical and Gynecological Association—The Pocket Pharmacy—Diseases of the Nervous System—Diseases of the Eye.
SELECTIONS AND ABSTRACTS—Itching in Scarlet Fever ................. 441
Strontium Lactate in Tænia ..................................................... 441
Eggs in Rectal Feeding ........................................................... 444
The Pathognomonic Signs of Perforating Appendicitis ................ 454
Milk: Is its Sterilizing Neces ary ............................................. 457
Chlorine in Combination with Phenic Acid—The Ideal Antiseptic and Disinfectant ..... 458
Treatment of Rheumatism ........................................................ 460
The Treatment of Gonorrhœa ................................................... 462
Injection of Alcohol in Uterine Carcinoma ................................. 463
Preventing Blindness ............................................................. 467
EDITORIAL—Observations upon Surgical Measures in Philadelphia ...... 464
The Tri-State Medica Association of Alabama, Georgia and Tennessee 466
Personals .............................................................................. 467
Books and Pamphlets received ................................................. 468
PRESCRIPTION DEPARTMANT ................................................. 471–472
Hay Fever—Diphtheria—Chronic Tonsilitis—Palpitation of the Heart—Meningitis—Bronchial Asthma—Asiatic Cholera—Hepatic Colic—Cholera Morbus of Malarial Fever—Fibroid Phthisis—Laryngeal Tuberculosis—Anemia
SPECIAL NOTES ....................................................................... 469–470

